# Revealing the Concealed in Monocular and Binocular Vision

**DOI:** 10.3390/vision9020047

**Published:** 2025-06-03

**Authors:** Nicholas J. Wade

**Affiliations:** Psychology, Nethergate, University of Dundee, Dundee DD1 4HN, UK; n.j.wade@dundee.ac.uk

**Keywords:** hidden images, contrast, stereoscopic depth, binocular rivalry

## Abstract

Concealing images has been a concern of artists and scientists, as have the conditions that can reveal them. It is relatively easy to hide images in pictures, but this is of little value if they remain hidden. The skill is in revealing previously concealed images. Three aspects of hiding images are examined, two of which are monocular and the third is binocular. Firstly, high-contrast patterns, like Street figures and Mooney faces, have been used in psychological tests of pattern recognition, and Gestalt grouping principles can result in concealing images. Second, it is possible to hide low spatial frequency content carried by high-spatial-frequency patterns. A wider range of carriers than gratings can be used, like graphics, photographs, and combinations of them (photo-graphics). Pictorial images can be concealed in terms of detection or recognition. In both cases, there is interplay between the global features of the concealed image and the local elements that carry it. Third, randomly textured stereograms reveal to two eyes what is concealed from each one alone—stereoscopic depth. The dimension of stereoscopic depth can be manipulated, as can that of binocular rivalry, to conceal images.

## 1. Introduction

**Figure 1:**
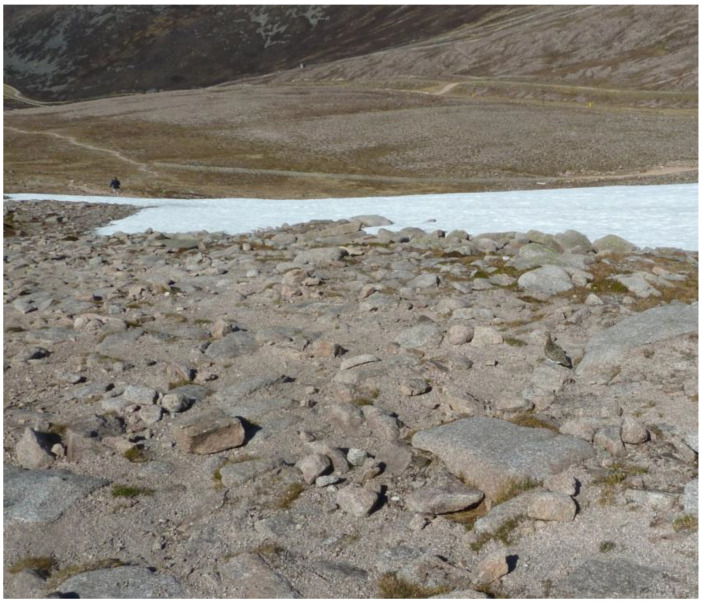
A photograph of a ptarmigan in the Cairngorms of Scotland. The patterning of the bird’s feathers makes it difficult to detect against the rocky terrain. The bird is located on the right hand side, below the band of snow.

In the natural world, camouflage has evolved as a form of concealment that has served adaptive purposes of evasion or detection by prey. If it is difficult to see an animal against its background, then, it is less likely to be hunted by a predator [1]. An example is shown in Figure 1.

**Figure 2:**
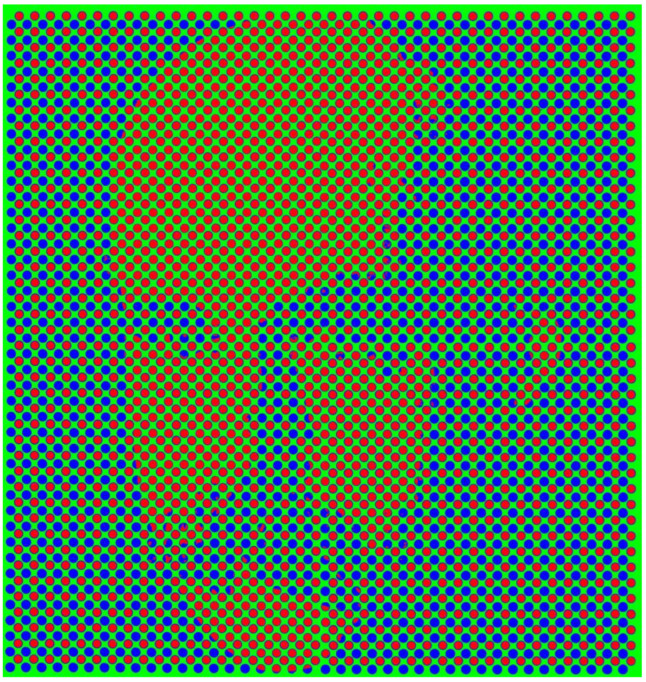
*Good Gestalt—Max Wertheimer* by Nicholas Wade. The dots defining Wertheimer’s portrait are either red or blue dots and the background is green. Not only is the portrait initially difficult to discern, but the uniformly coloured regions are modified by their surrounds.

Similarly, images can be concealed graphically in order to evade detection or to disrupt recognition [2]. It can be difficult either to detect the presence of a particular component in a pictorial image or to determine the identity of the concealed element. For example, it is possible to detect the presence of a face without recognizing who it is a representation of. There are various ways in which images can be concealed, or more precisely, made harder to detect or recognise. Images can be carried in some pattern or design, such that minor variations in the design define features that are not initially detected; they can share contours so that the interpretation is ambiguous; the viewpoint can be distorted; and they can be presented in contrasts or orientations that hide details normally visible. The illustrations to be shown often require some effort on the part of the viewer to discern the figures embedded in them (Figure 2).

Pictorial images can be concealed in terms of detecting the patterns in their context or of recognising their identity. In both cases, there is interplay between the global features of the concealed image and the local elements that carry it. This type of concealment is a relatively recent addition to the armoury of devices available to picture makers and it can be more readily achieved by means of photography or computer graphics. It is with such concealments in monocular and binocular vision to which the following illustrations speak.

## 2. Contrast

**Figure 3:**
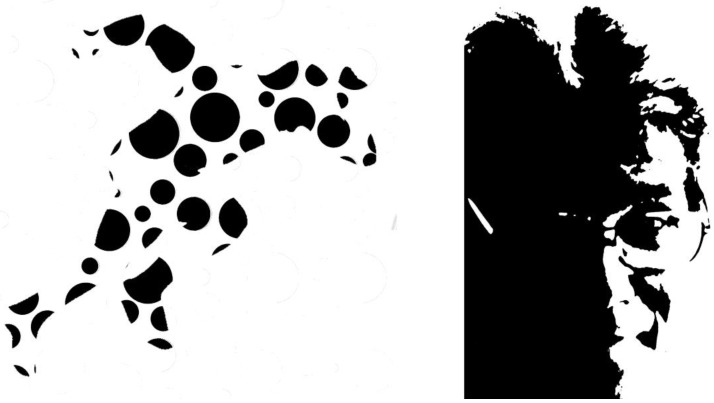
Illustrations based on the techniques used to make Street and Mooney figures. (**Left**) *Running man*, and (**right**) *Amodal Kanisza* (both by Nicholas Wade). The outline of the running man is defined by the phenomenon of subjective contours so subtly manipulated by Gaetano Kanisza.

Powerful contrasts can be produced with black and white patterns as well as those with contrasting colours. Both the Gestalt Completion test [3] and the Mooney face test [4] utilise high-contrast versions of graphics or photographs in order to render recognition difficult. Fragments of high-contrast images are employed in the Street test and the Mooney test presents faces in stark areas of black and white. Figure 3 shows illustrations based on these techniques.

Street figures and Mooney faces are both based on the older technique of silhouettes and positive/negative reversals. Silhouettes of faces were typically made from the only direction for which there is no ambiguity—the profile view. This has been described as the stereotypical viewpoint as it involves the minimal foreshortening of the asymmetrical axis of the face [5].

**Figure 4:**
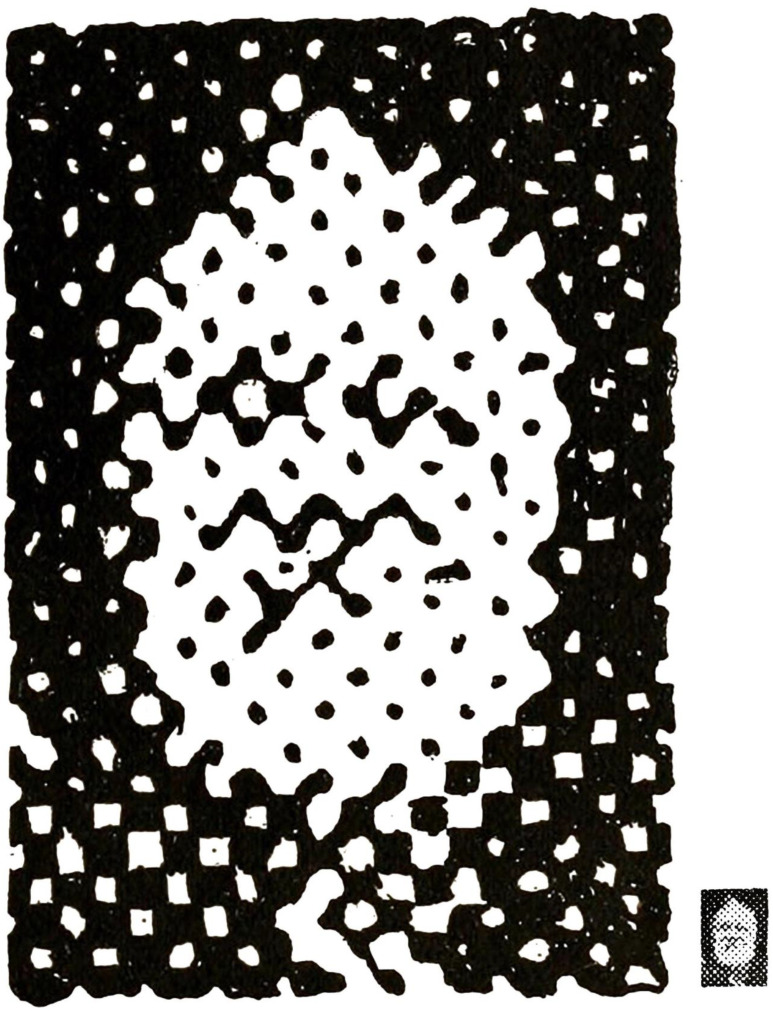
Detail of a photomechanically printed portrait of Lord Kelvin (Figure 7 from Jastrow [6]) together with a small version of it.

Half-tone images used in photomechanical printing are grey-scale images that have been filtered by a high-spatial-frequency black-and-white dot screen. The intensity of the original image modifies the sizes of the dots so that the picture appears to contain greys rather than just black-and-white. Since the dots cannot be resolved, the brightness averaging creates the impression of shades of greys rather than black and white. If small areas of the half-tone image are enlarged then the greys disappear and the object imaged can be difficult to recognise. Jastrow [6] provided such an example, which is shown in Figure 4, but in small scale and enlarged (although Jastrow only illustrated the enlarged version). In the case of Kelvin’s image, the small dots have been enlarged as described by Jastrow: “a highly enlarged reproduction taken from a half-tone process print” [6] (p. 305). Jastrow appreciated how the concealed image could be revealed: “A curious optical effect, which in part illustrates the change in appearance under different aspects, is reproduced in [his] Figure 7. In this case the enchantment of distance is necessary to produce the transformation. Viewed at the usual reading distance, we see nothing but an irregular and meaningless assemblage of black and white blotches. At a distance of not less than fifteen to eighteen feet, however, a man’s head appears quite clearly. Also observe that after the head has once been realized it becomes possible to obtain suggestions of it at nearer distances” [6] (pp. 306–307). This strategy will apply to many of the pictorial images that follow.

### High Contrast Patterns

**Figure 5:**
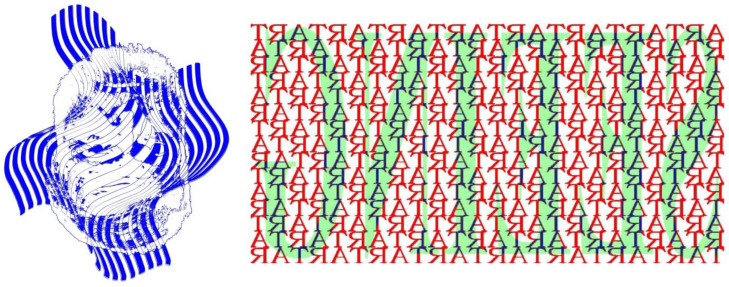
High-contrast images that are difficult to recognise because of spatial inversion and reversal. (**Left**) *Hemingwaves II*, and (**right**) *Seeing Art* (both by Nicholas Wade).

Gestalt principles of perceptual organisation can operate to conceal as well as reveal. This applies to figure-ground and interpretative ambiguities, too [7]. Alternative interpretations can be made difficult to recognise, and this has been an artistic enterprise for centuries [8]. Elements of patterns can be grouped in many ways, according to the principles formulated by Wertheimer [9,10]. In general, the principles result in the emergence of a “whole” from the “parts” of a global organisation. Gestalt grouping principles can be used to render recognition difficult. Inversions and reversals can conceal recognizing high-contrast images. Inversion adversely affects recognition not only of faces but also of text, as Rock [11] remarked. Figure 5 presents two examples: on the left, the identity of the famous portrayed person is disrupted because of inversion; on the right, the reversal of text and the absence of spaces makes the background pattern difficult to read and the lager, low contrast letters almost illegible. Inverting the figure will restore the identity of the portrayed person (the name of whom is indicated in its title) and reversing it will render both words legible.

**Figure 6:**
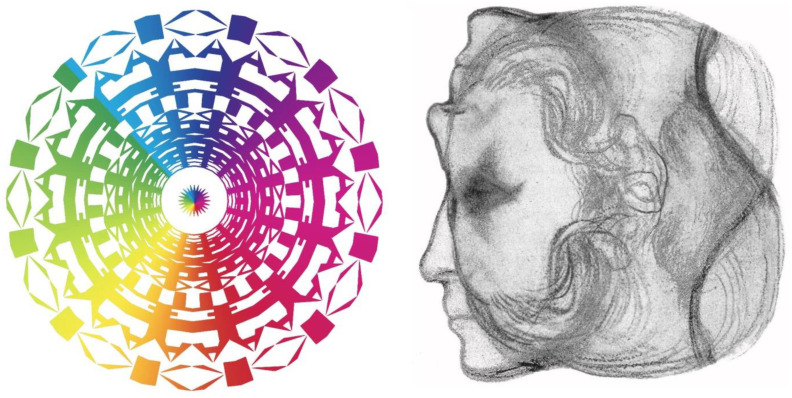
(**Left**) *Kaleidoscopic kaleidoscope*, and (**right**) *The two faces of Rex Whistler* (both by Nicholas Wade).

Gestalt grouping can be in terms of symmetry as well as orientation. Orientation is a fundamental feature of Gestalt grouping that is often overlooked. The positioning of local and global features, with respect to the observer or with respect to gravity, provides a signal source of segregation and recognition. Reflection and rotation are the fundamental aspects of kaleidoscopic images, and they have been applied to the word itself (Figure 6 left). Symmetry and inversion are features of a long-standing artistic genre involved in displaying two faces in one. Profile views of faces have most commonly been employed and examples can be found in Roman pottery and mosaics [7,12,13]. The eyes are used as the line of horizontal symmetry in most works, but the mouth has also been the common feature. Rex Whistler [14,15] is remembered for his witty representations of two faces in a single picture. The two faces typically share eyes, but the inverted one is generally unrecognisable until the design is itself inverted. It is surprising that Whistler did not include variations with a mouth common to both upright and inverted heads because the patterning of the open lips defines a positive or negative expression of a face [16]. Nonetheless, a feature that he added to the upside-down genre of visual trickery was the combination of jovial and jaundiced expressions in the same drawing. This was achieved not only by manipulating the shape of the mouth but also by the location of the eyes in the orbits: the unhappy face is generally accompanied by upwardly directed eyes, with the reverse for the happy face. The location of the eyes also influences the conformation of the brows, which is an additional facial correlate of the two expressions. His upright and inverted portrait is shown in Figure 6, right.

## 3. High- and Low-Spatial-Frequency Interactions

Interactions between the high- and low-spatial-frequency content of displays became of interest in visual science after Harmon [17] displayed a quantised portrait of a human face: “Another approach to human recognition of human faces is to ask how little information (in the informal sense of binary digits) is required to represent, pictorially, a face to be recognized out of the finite ensemble of faces. One way to explore this is to use precisely blurred portraits. With computer-produced image degradation, an exact measure of blurring (2-dimensional low-pass spatial filtering) is possible, unlike optical defocusing which cannot be explicitly specified and controlled with great precision” [17] (p. 206). A grey-scale picture was broken down to a 16 × 16 matrix of blocks in which the luminances are averaged, making recognition of the face difficult. The technique was made popular after its description by Harmon and Julesz [18] in *Scientific American* using a portrait of Abraham Lincoln. It was rapidly adopted in art with Dalí’s *Gala contemplating the Mediterranean sea which at twenty metres becomes a portrait of Abraham Lincoln* painted in 1976 (see Bach’s demonstration at https://michaelbach.de/ot/fcs-mosaic/ accessed on 20 March 2025). Dalí’s awareness of developments in visual science is evident in the painting and its title. He even provided a painted version of the Lincoln image as one of the blocks in his larger work. At Figueras, where Dalí’s museum is located, this large painting can be viewed through a defocussing lens so that Lincoln’s portrait emerges more prominently. As Braddick et al. [19] noted with regard to Harmon’s Lincoln: “A variety of other displays may be devised in which high spatial frequencies apparently mask much lower frequencies in similar fashion” (p. 33). Despite the precision of spatial filtering by computer, many of the illustrations presented in the following pages have not been produced using a computer.

**Figure 7:**
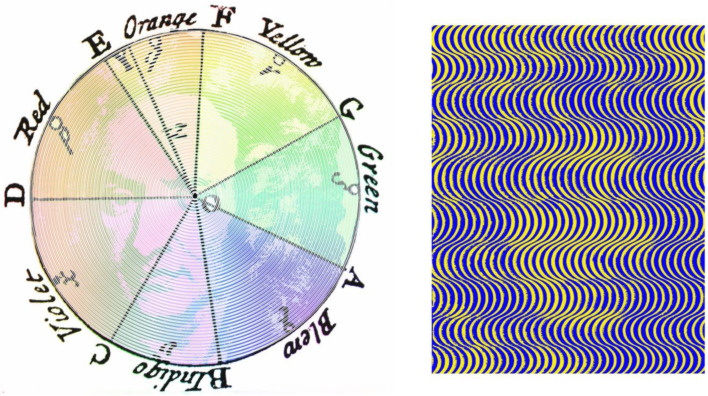
(**Left**) *Newton’s colour circle*, and (**right**) *Huygens’ waves* by Nicholas Wade. Newton’s left eye is at the centre of his diagram of the colour circle portrait and Huygens can be seen in a pattern of waves.

Pictorial images can be carried in some pattern or design, such that minor variations in the design define features that are not initially visible. As in the case of Harmon’s digitised images, faces have proved to be the most popular stimuli to subject to such concealment. Figure 7 shows two examples of what I call “perceptual portraits”—they represent people in an unconventional style often hiding their appearance, as was the case in several of the figures above. Perceptual portraits typically consist of at least two elements—the portrait and some appropriate motif. The nature of the latter depends upon the endeavours for which the portrayed person was known. In other perceptual portraits, the motif is derived from a figure or text in one of their books, or apparatus that they invented. The portraits and motifs have themselves been manipulated in a variety of ways, using graphical, photographical, and computer-graphical procedures. The two portraits in Figure 7 are of Newton and Huygens, both of whom extended the understanding of the nature of light and vision. Newton’s corpuscular theory of light was opposed by Huygens’ wave theory. In the context of colour, Newton proposed three primary colours (in the red, green, and blue parts of the spectrum), whereas the primaries were blue and yellow for Huygens.

**Figure 8:**
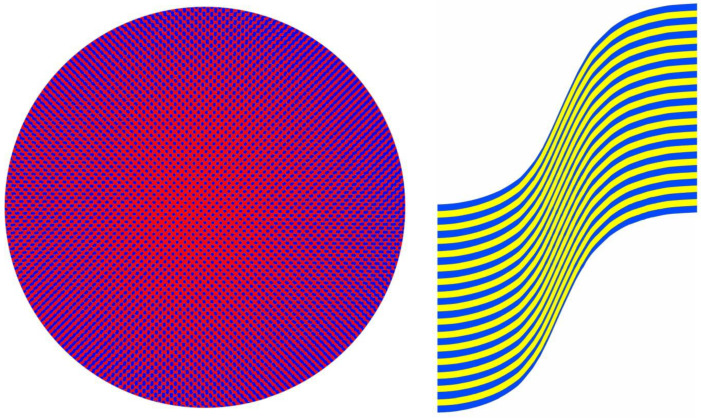
(**Left**) *Berkeley’s distance*, and (**right**) *Fechner’s functions*, both by Nicholas Wade.

Figure 8 presents perceptual portraits of two towering visionaries who have influenced views of how we see and how we measure what we see. Berkeley [20] was an empiricist philosopher who argued that appearances are all: existence is perception. He stated that distance cannot be directly perceived as a line extending from the centre of the eye is perceived as a dot. The pattern of dots in which his portrait is embedded represent extended lines only one of which is directed to the centre of his left eye. Fechner [21] considered that the mental and material worlds could be united mathematically in the domains of stimulus and sensory intensity—what he called psychophysics. He is portrayed in a series of curves representing psychophysical functions, and his dimly defined profile is to be found in the part of the function called the area of uncertainty.

High-contrast, high-spatial-frequency contours can suppress the visibility of low-contrast, low-spatial-frequency components within them [19,22]. The concealed portrait can be revealed in a number of ways, all of which involve reducing the visibility of the high-spatial-frequency content. The techniques include the following: removing spectacles, if they are worn, or defocusing the pattern by accommodating to a point nearer to or further from the picture plane; viewing the picture from such a distance that the details are no longer sharply resolved or reducing the size of the image; and moving the head or the image rapidly from side-to-side.

### Distractors

**Figure 9:**
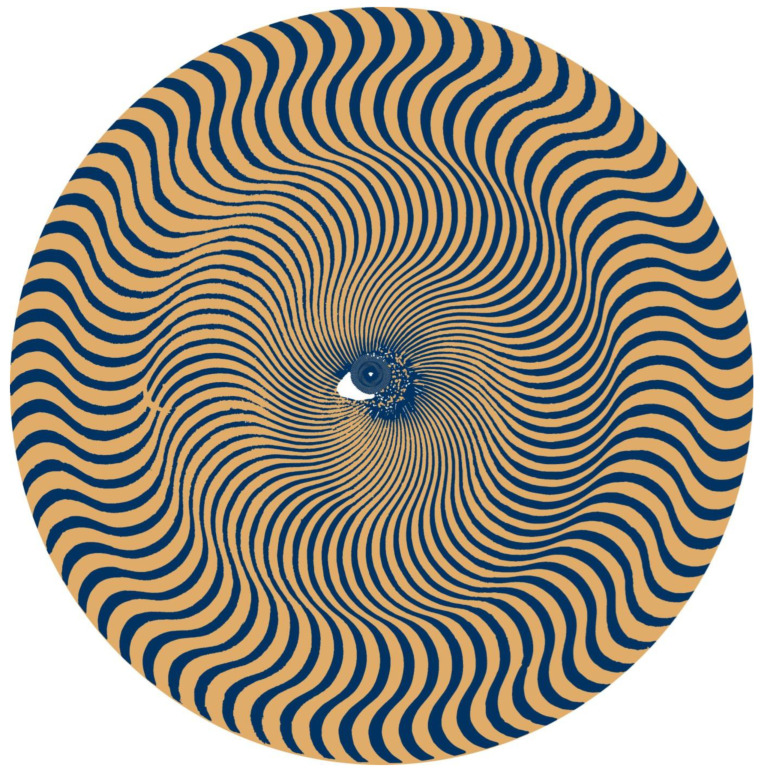
*Chrys-anthem II* by Nicholas Wade.

Content can be concealed either partially or completely by presenting clearly visible distractors in the pictorial image. The distractors direct attention away from the more subtle pattern variations that carry the partially hidden content or broad-band distractors can mask low-contrast components. Figure 9 is a puzzle in several senses. Firstly, the pattern of radiating curves is itself enigmatic because it can be interpreted as an impossible figure—contours that appear as humps on one side seem to be hollows on the other. The distractor is a clearly visible eye in the centre of the pattern, but this eye is itself defined by the same radiating pattern, but at a smaller scale and at lower contrast. The left eye is so well-defined that a search for its partner is not pursued. There is not only a right eye on the left of the pattern but also a whole face with nose, lips, chin, and flowing hair. The radiating lines surrounding the central eye follow the contours of the nose and orbit.

**Figure 10:**
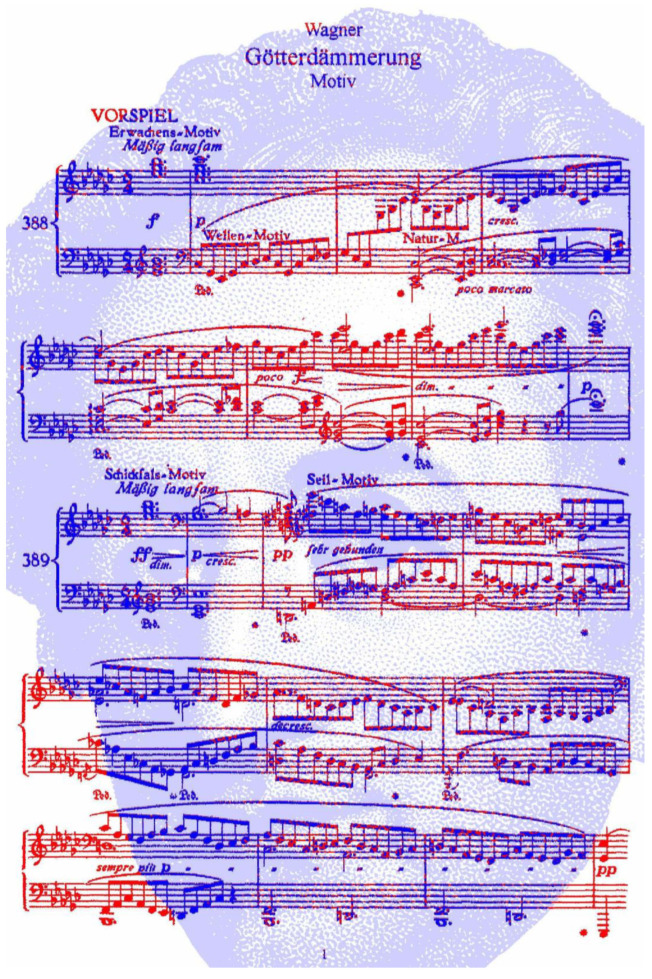
*Goethe’s Dämmerung II* by Nicholas Wade.

Distractors can also dominate the perception of a pattern so that image remains concealed for longer, particularly if it is fragmented, as in Figure 10. The musical score of the opening to the overture for Richard Wagner’s opera *Götterdämmerung* (Twilight of the Gods) carries with it a portrait of Wolfgang Goethe in the blue notes. The image needs to be viewed from a distance or reduced in size before the concealed image is revealed. As with most monocularly concealed images, once revealed, it can be more readily seen subsequently.

A link can be made between monocular and binocular concealed images by utilising a variation in the wallpaper illusion. It was described before the invention of the stereoscope by Wheatstone [23] in the 1830s, but its significance was not appreciated until after that [24,25,26]. It can be seen in patterns that consist of horizontal and vertical repetitions, like the flowers on wallpaper, and it was illustrated with such a pattern by Brewster [27] in 1844. When the same element is fixated with both eyes, then, the whole pattern appears to lie in the plane of the wallpaper. When equivalent but laterally separated patterns are combined binocularly, they seem suspended in the plane of convergence. The depth at which the pattern is seen corresponds to the plane at which the eyes converge: the farther apart the combined elements are the greater the apparent depth. The wallpaper illusion provides the basis for illustrations referred to as autostereograms. Rather than seeing a single-depth plane, multiple-depth planes can be seen. Wallpaper illusions and autostereograms can be seen without the aid of any viewing device; they involve dissociating convergence from accommodation by converging the eyes to combine neighbouring elements or viewing them with parallel visual axes.

**Figure 11:**
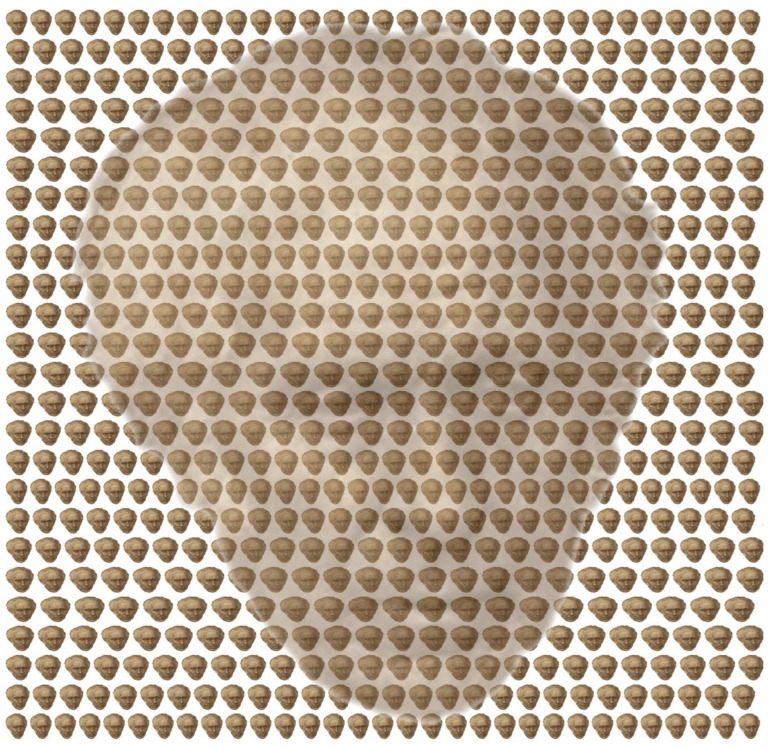
*Wallpaper Brewster* by Nicholas Wade.

In the 1990s, algorithms for generating autostereograms with computers made them enormously popular. In large part, this was because a viewing device was not necessary in order to experience the stereo effects, although some people do find it difficult to converge their eyes appropriately to maintain that degree of convergence and to dissociate accommodation from convergence. If slight variations in the widths and locations of the repetitive elements along rows are introduced (as in Figure 11), then more complex depth planes are visible and aspects of disparity processing become involved. The surface no longer looks flat but stepped in wedges from top to bottom. Figure 11 is a simple version of an autostereogram; they usually hide images that can be recognised once the depth planes have been differentiated: depth is concealed before the images are revealed. If slight variations in the locations of the repetitions along rows are introduced, then, more complex depth planes are visible and aspects of disparity processing become involved. This is the principle employed in autostereograms [28].

In addition to the stereoscopic depth that can be seen in Figure 11, there is a monocular concealment, too. The pattern is made up from an array of small portraits of Brewster that vary systematically in size and separation from top to bottom. When viewed from a distance the detail of the small portraits is lost and an enlarged version of Brewster’s portrait becomes visible.

## 4. Binocular Vision

The various methods of concealment displayed so far can be revealed to a single eye, but this is not the case for the illustrations to follow. This should not be a surprise because the vast majority of the pictures we see are made for one eye and not two. Although we look at these pictures with two eyes, there are very few that are made to be seen so. That is, very few pictures require binocular vision to see the features they contain. The two eyes can cooperate to yield stereoscopic depth and they can compete with one another, resulting in rivalry. Of course, the cooperation or competition is not between the eyes themselves, but the signals they send to the visual brain. Each aspect will be presented separately, although, in natural binocular vision in a three-dimensional space, both processes are constantly in operation.

There are several ways in which paired pictures can be viewed to yield stereoscopic depth. The simplest is free viewing, where adjacent pictures, equivalent to what is seen by the left and right eyes, are superimposed by adjusting the directions in which the eyes are pointing; with parallel viewing, the left pattern is seen by the left eye and the right pattern by the right eye, whereas with crossed convergence the reverse occurs. Wheatstone [23] was able to view his stereograms in this way, but he found that many others to whom he showed them could not. It was for them that he invented the stereoscope that made the superimposition of the half-images much easier. The model he described in his paper of 1838 was a reflecting (mirror) stereoscope, but he had constructed models based on refraction by prisms before that [24]. Anaglyphic viewing involving colour separation was introduced later, and it is this form that will be presented below, together with the left and right eye images; the left and centre images are for parallel viewing and the centre and right images for crossed convergence. The arrangement is referred to as Universal Freeview, and the dots above to centres of the images are to aid alignment. Anaglyphs require red/cyan viewers that are matched to the displayed colours; each half-image is blocked by one colour filter and passed by the other. They are simple stereoscopes.

### 4.1. Stereoscopic Depth Perception

**Figure 12:**
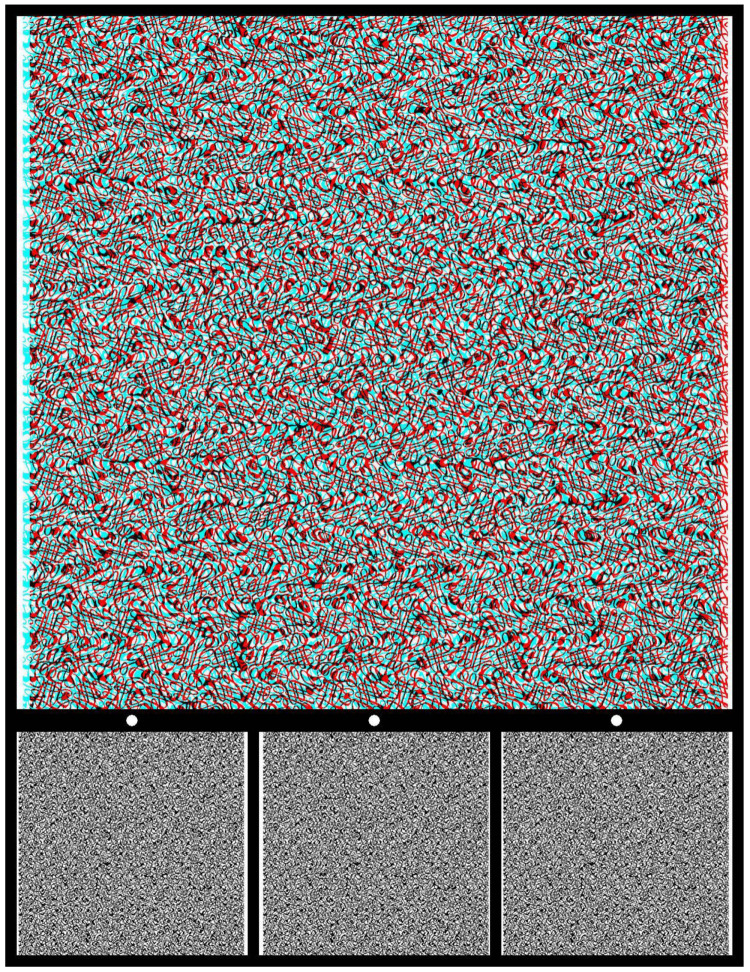
*Disc in depth* by Nicholas Wade. An anaglyph in which a disc is seen in depth when viewed through red/cyan glasses; with the red filter in front of the left (L) eye, and cyan filter in front of the right (R) eye, the disc appears more distant than the surround. Reversing the filter/eye arrangement reverses the stereoscopic depths. Lower: Universal Freeview arrangement of the monocular images in the sequence L-R-L. There are two ways to fuse the two stereo sets of images so as to see depth: parallel or uncrossed viewing for the left pair or fusing the right pair with crossed-eye viewing. The dots above monocular half-images can be of assistance in alignment. The enclosed disc appears in the opposite depth when the alternative monocular images are combined. The two half-images are displaced by equal amounts left and right as would be the case for binocular photographs taken using Cajal’s method. Since the monocular components are much smaller than the anaglyphs above them, it might be necessary to enlarge the images in order to extract the details for free fusion.

Binocular depth is another aspect of concealment that has been employed in the past. What two eyes reveal that is concealed from each eye alone is stereoscopic depth. One of the earliest random-textured stereograms was made by Ramón y Cajal in 1870 when he was still a medical student [29,30]. He later described his method of concealment: “My aim was to achieve a mysterious writing, which could only be deciphered with the stereoscope and usable for those people who don’t want to divulge their own matters… The game consists of making a proof [a print on glass] only with dots, lines and scribbles, or also of letters, crossed and entangles in a thousand ways. A proof in which, with the naked eye, you cannot read anything at all. And, nevertheless, as soon as you see the double image of this background in the stereoscope, a perfect legible sentence or text suddenly appears, standing out on the foreground and clearly detaching itself from the chaos of the lines or dots” [29] (p. 71). The separated patterns on glass and board were photographed with a binocular camera and the two half-images were viewed in a stereoscope. By using a binocular camera and viewing the photographs in a stereoscope, Cajal created disparities in both half-images relative to their common backgrounds. An anaglyph analogous to this is illustrated in Figure 12. The carrier pattern is made up of dense squiggles not unlike those described by Cajal.

Random-dot stereograms [31,32] hide the patterns they contain if only one eye is used, but they can be revealed with binocular observation. Julesz wished to study stereoscopic depth perception without any knowledge of the objects that were to appear in depth. A precursor of a random-dot stereogram can be found in Wheatstone’s [23] article describing the first stereoscope: a row of five dots was presented to each eye, but those in one eye had larger separations than in the other. If the horizontal rows had been extended vertically then a surface slanting in depth would be visible stereoscopically [30]. With the advent of computer-generated images, Julesz made random-dot stereograms in which there was nothing presented to either eye alone that could indicate the depth to be seen when the two images are fused. Only with their combination could the depth emerge in what Julesz called cyclopean vision. As noted above, before Julesz’s technique was devised, Cajal developed a stereoscopic method for concealing images. It is not unlike the principle of random-dot stereograms and had the intention of encrypting messages.

**Figure 13:**
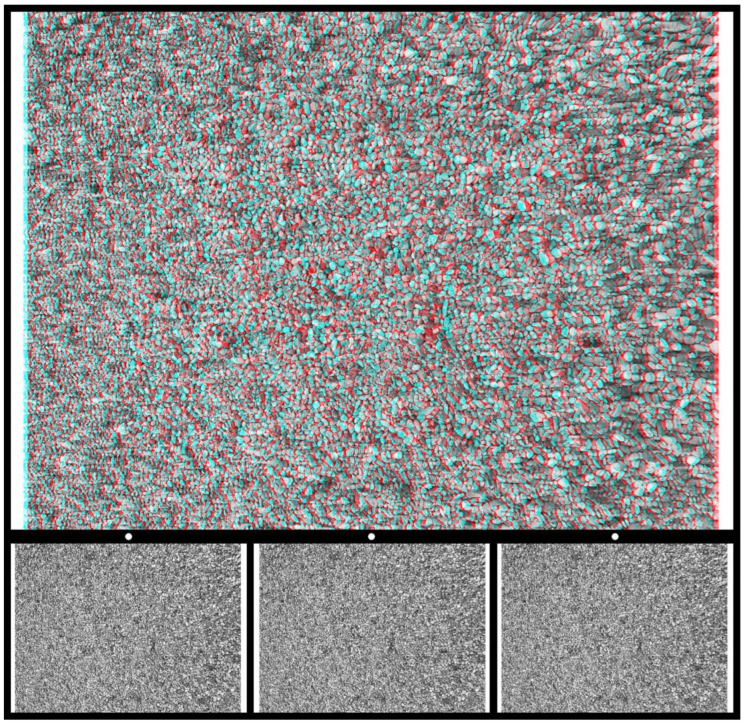
*Quadrilaterals* by Nicholas Wade.

The patterns for the disparate monocular half-images can be more complex than dots and squiggles as can the concealed shapes [26,33]. The textured carrier patterns can be based on graphics, photographs, or combinations of them (photo-graphics). Figure 13 uses a photograph of stones in which two rectangles are seen at different depths when the left and right eye images are combined. With the red/L and cyan/R arrangement, the smaller central one appears nearer than the larger rectangle surrounding it and both look nearer than the background. Not only does the depth reverse with cyan/L and red/R, but the quadrilaterals also look larger.

**Figure 14:**
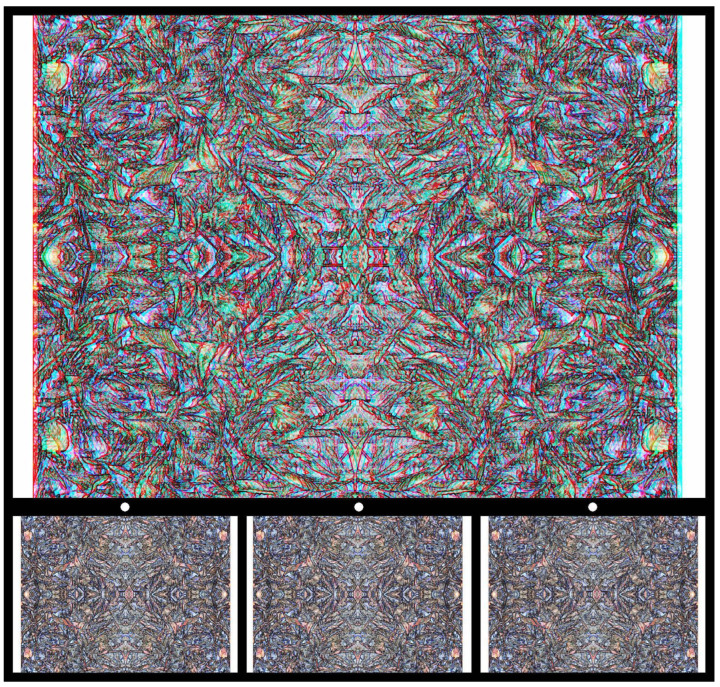
*These are not tobacco leaves II. Homage to Magritte* by Nicholas Wade.

More elaborate carrier patterns can be constructed when graphics (drawings and paintings) are combined with photographs prior to introducing disparities between them [26,33]. In Figure 14, the original photograph of dried tobacco leaves is the manipulated photographically so that its origins are finally obscure. The photograph was quadruplicated to give it a vertical and horizontal symmetry; a line image was then made from it. Only at that stage was a pipe shape extracted and displaced laterally in one half image before its combination. Viewing it stereoscopically results in the appearance of a pipe either hovering in front of the background or seen through it. The pipe shape is a reference to Magritte’s painting of a pipe above the painted words that it was not a pipe. The title of Figure 14 is a homage to Magritte, but, in this case, the reference is made to the tobacco leaves rather than the pipe. The tobacco leaves cannot be recognised with either monocular or binocular viewing, whereas the pipe is revealed stereoscopically.

**Figure 15:**
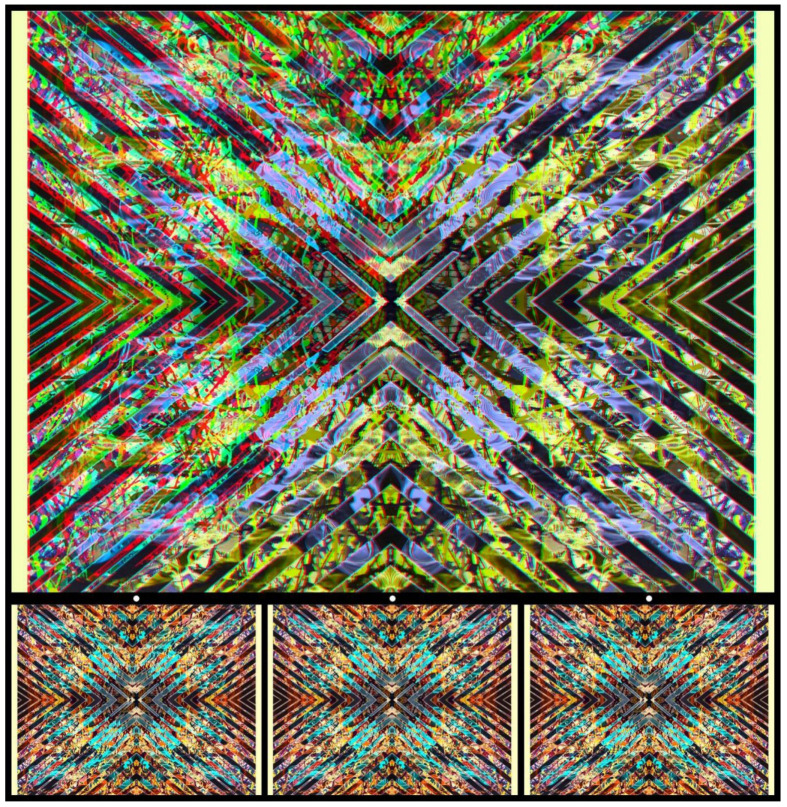
*Sigmoid surface* by Nicholas Wade.

For Figure 15, the combination is of two paintings in quite different styles. Detail from an abstract tachiste painting is intertwined with the contours of a more constrained geometrical design. The combination was then further manipulated with computer graphics. A sigmoid function divides the whole surface; it is not visible to either eye but stands out strikingly in stereoscopic view. With red/L and cyan/R or with parallel viewing, the right-hand side appears closer than the left.

**Figure 16:**
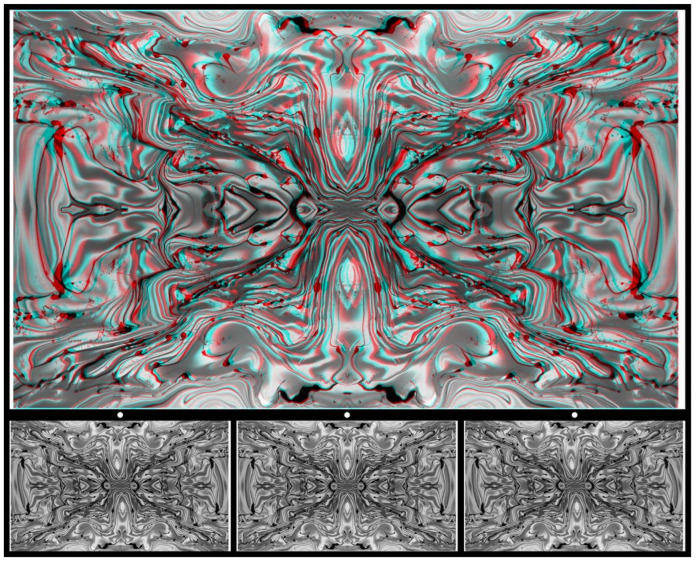
*Sign wave* by Nicholas Wade.

A distinction between Figure 12, Figure 13 and Figure 14 and Figure 15 is that the regions that are hidden from one eye but revealed to two are enclosed within the patterns, whereas, in Figure 15, two surfaces are separated in depth. This textured surface stereoscopy can be demonstrated in isolation, as in Figure 16. The initial flow painting was photographed, quadruplicated, and rendered black-and-white. One half-image was distorted and the other was not before a stereoscopic combination was composed. With stereoscopic viewing, the depth takes a little longer than in Figure 12, Figure 13 and Figure 14 and appears to increase with time so that a deep horizontal wave covers the whole space. The trough is in the upper portion of the image with the peak beneath it with red/L and cyan/R or uncrossed free viewing. The peak replaces the trough with the alternative viewing arrangement.

### 4.2. Binocular Rivalry

Stereoscopic depth perception is a consequence of binocular cooperation, whereas rivalry reflects binocular competition [34]. Binocular rivalry is a natural consequence of our binocular interactions with the world; it is a resolution of conditions that apply to most of what we see when using two eyes. It occurs when the differences between the images in the two eyes are too large to be combined. When we fixate with both eyes on an object, most of what is projected to the peripheral retina is too disparate to yield depth; since the peripheral stimuli tend to arise from different depths to those fixated, their retinal images also tend to be out of focus. We are not generally aware of this binocular rivalry as both visual resolution and attention are associated with the fixated object rather than peripheral ones. Binocular rivalry is rarely examined under these conditions of natural stimulation. It is typically studied with different patterns presented to corresponding foveal regions of the two eyes—as if we are fixating on two different objects at the same time. Under these conditions, our vision is unstable, and it is this state that has been the subject of much scientific enquiry, but it has resulted in little art.

**Figure 17:**
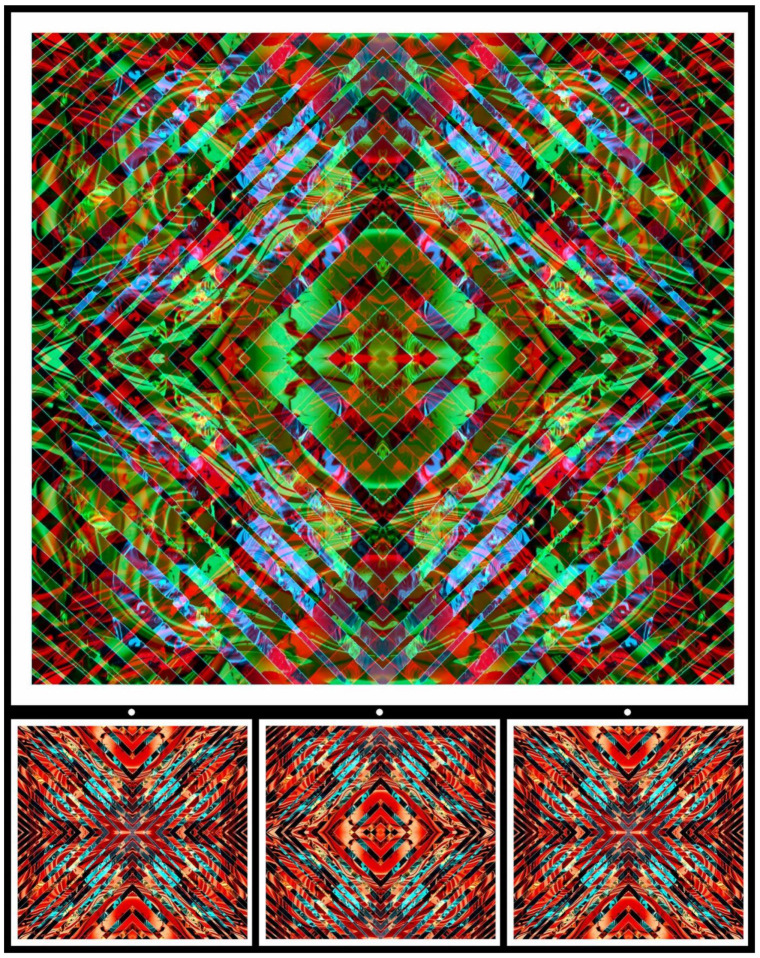
*Orthogonality* by Nicholas Wade.

Stereoscopic depth is seen in stereograms when viewing patterns in each eye that contain large regions of correspondence and smaller, defined regions of slight and systematic non-correspondences. To produce rivalry, each eye is presented with large non-correspondences so that fusion is not possible. Concealing and revealing take on different complexions in binocular rivalry because it is never clear what parts of the monocular stimuli are concealed and revealed. Rivalry is a dynamic process and it is characterised by change, as can be seen in Figure 17. The patterns perceived are mostly mosaics made up from fragments of the two monocular stimuli. The visibility of the pattern presented to one eye is rarely seen and then only fleetingly. Rivalry is also a phenomenon in which eye dominance plays a more noticeable role than in stereopsis.

**Figure 18:**
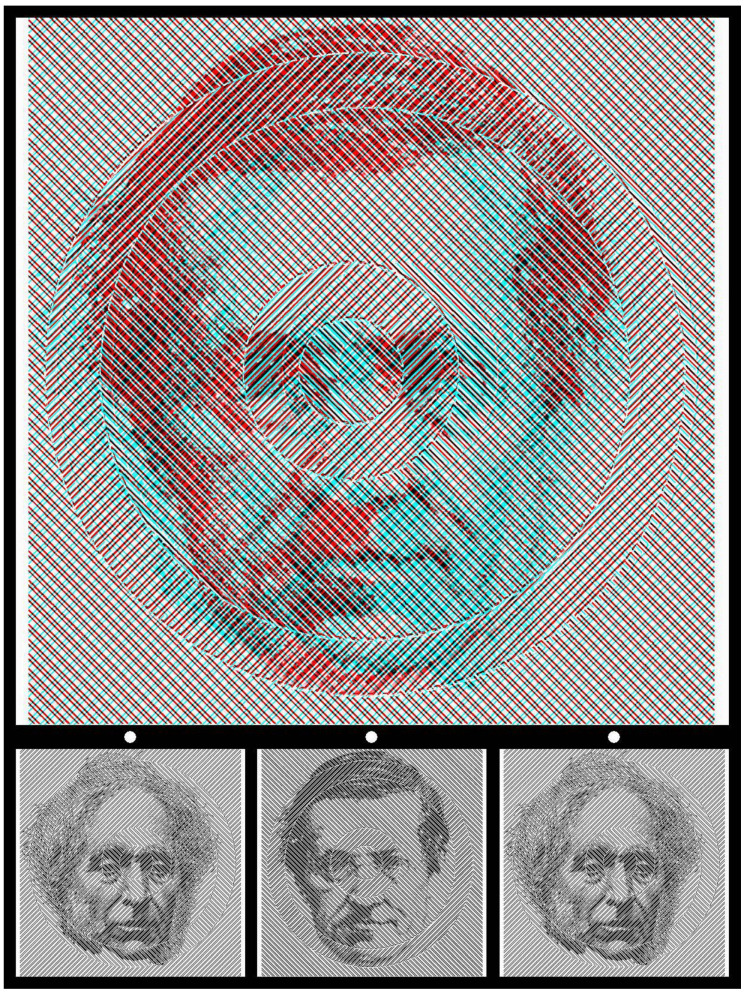
*Rivalling Brewster and Wheatstone* by Nicholas Wade.

More subtle variations in rivalry occur by adapting some of the monocular methods of concealing in one of the half-images of a stereogram. For example, a portrait can be partially hidden in a design that engages in rivalry with another pattern. Moreover, the patterns used for rivalry can reflect the endeavours for which those portrayed are associated. Two pioneers of stereoscopy, Charles Wheatstone and David Brewster, examined binocular rivalry, although they engaged in a bitter personal rivalry, too [24,35]. They are shown within a rivalling background in Figure 18, in addition to their rivalling portraits.

**Figure 19:**
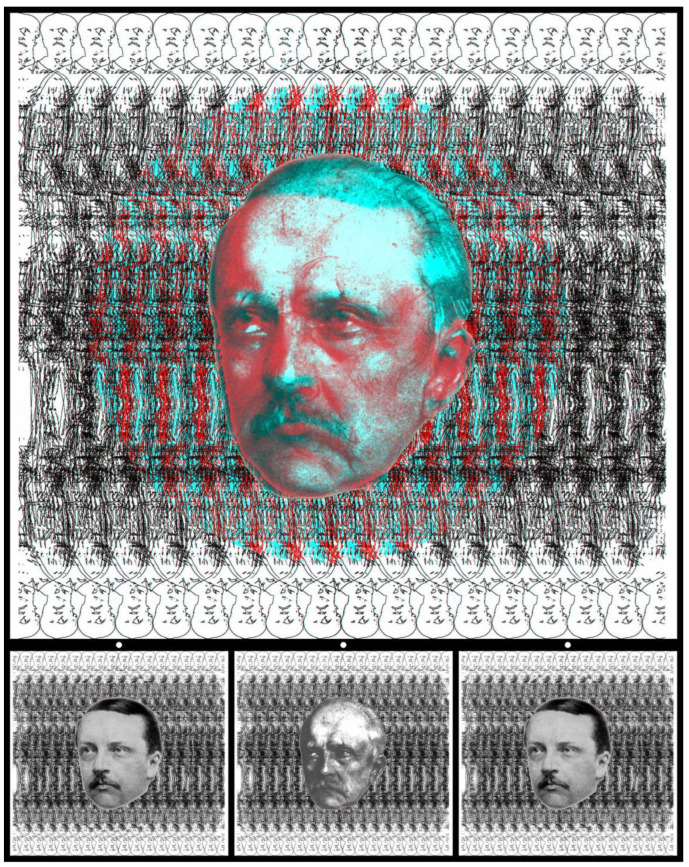
Helmholtz in rivalry and depth by Nicholas Wade.

Rivalry and stereopsis can coexist, and this aspect is displayed in Figure 19. Rather than portraying different individuals in rivalry, the same individual can be shown at different ages. This is possible if many portraits of the same person are available, be they engravings, paintings, or photographs. There are many portraits of Hermann Helmholtz and selections can be made from those having the same head orientation, making combination possible. Helmholtz carried out extensive investigations into binocular vision and these included rivalry. However, in Figure 19, rivalry is restricted to the portraits, and the patterned background can be seen in stereoscopic depth. The apparent depth remains during the phases of rivalry between the portraits. The portrayals are from 1857 and 1894 when Helmholtz was 36 and 73 years old [36]. The younger Helmholtz is based on a photograph and the older one on a drawing. During binocular viewing, the portraits are in rivalry with one another but they are set in the centre of a circle that is in stereoscopic depth. The carrier pattern is derived from a photograph of a statue of Helmholtz that stands outside the Humboldt University in Berlin. The form of the statue has been multiplied and rendered in line form; outlines of Helmholtz’s head can be seen at the top and bottom of the figure.

**Figure 20:**
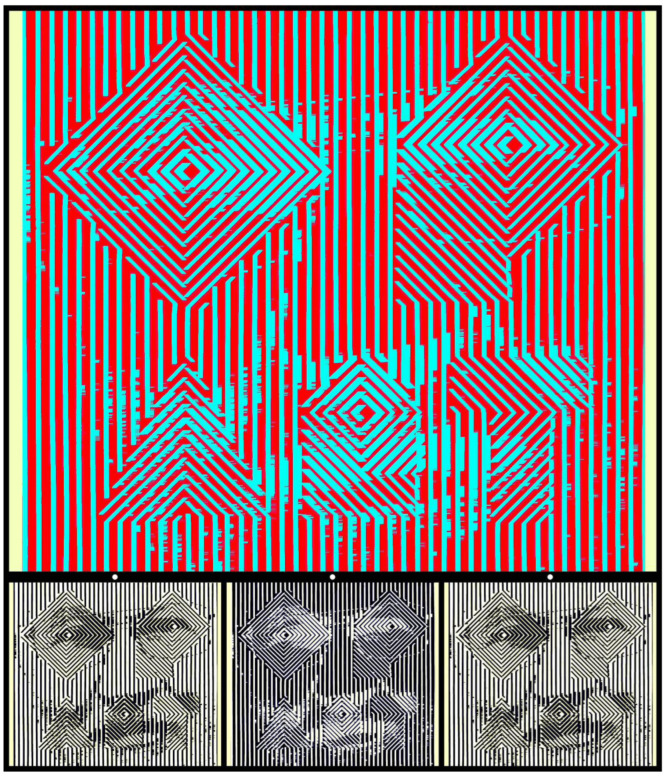
*Self portrait as an Op Artist* by Nicholas Wade.

The final image illustrates an aspect of binocular competition that has not received the same attention as contour rivalry—binocular lustre. It is so named because the combinations of a positive to one eye and a negative of the same image to the other create the impression of metallic lustre that is not in either image alone [36,37]. Three puzzles are presented in Figure 20. One concerns reading words within it and another is a portrait that is carried by it. The third puzzle is binocular; the figure presents a positive to one eye and a negative to the other. The words are defined by idiosyncratic letter shapes that speak to the enterprise that is embraced by them; the components of the face are readily detectable but they are presented at a spatial scale that initially conceals their recognition. The face is easier to see in the smaller monocular images beneath the anaglyph, and it is easier to see in the positive than the negative.

## 5. Discussion

The illustrations presented above demonstrate a variety of ways in which initially concealed elements in pictorial images can be rendered visible. The techniques draw upon many perceptual processes, some of which can be seen with one eye and others require two eyes. Pictorial images entered into visual science rather late. In the case of colour, it was freed from its object base by Newton [38]; prior to that, most observations were related to coloured objects rather than coloured light. In the case of space, its experimental investigations were transformed by instrumental ingenuities introduced in the early nineteenth century: Wheatstone [23] invented the stereoscope for examining apparent depth seen in two slightly different pictures and stroboscopic discs were devised to produce apparent motion in a sequence of slightly different pictures, each of which was briefly visible [25,39,40]. At around the same time, photography was invented by Daguerre [41] and Talbot [42]. Thereafter, experiments on vision tended to use pictorial stimuli. It is strange that objects are rarely studied in the area referred to as object recognition: pictures take their place. This applies to recent approaches that enlist the armoury of brain imaging techniques [43]. Object recognition is becoming restricted to a single object—the computer monitor. On the other hand, picture concealment is becoming a topic of great applied interest in a world awash with digital communication the security of which can be essential [44].

By contrast, artists have been engaged in pictorial concealment for millennia. Indeed, the very nature of the artistic enterprise involves concealment because pictures represent states that are not present. The marks on a cave wall might be recognised as an animal, but it is not one that can move or be eaten. With the introduction of more accurate methods of spatial representation (like linear perspective), artists were quick to find ways in which the rules could be manipulated (as in anamorphoses) so that pictures had to be viewed askew or with the aid of some instrument (like a cylindrical mirror) for the space to be recognizable [45,46]. Long before this, spatial ambiguities were systematically varied in Roman mosaics and the precursors of Gestalt grouping principles were displayed rather than described [13,47]. The standard ambiguities illustrated and examined in visual science, like Necker cubes and Rubin’s vase/faces [48,49], are simple in comparison to those in visual art [50,51], although there are exceptions [52]. The simplicity is a consequence of adopting scientific methods to determine the factors involved in their occurrence [53].

The science and art of concealed images can be in closer harmony, and that is an aim of the present article. The phenomena that have emerged from scientific scrutiny can be presented with an artistic flourish. The three types of image concealment considered—high-contrast patterns, high- and low-spatial-frequency interactions, and binocular cooperation and competition—continue to attract scientific as well as artistic attention. Mooney figures can be produced more easily with the use of lith film in high-contrast photography [5,54] and with thresholding in computer graphics. Variants on Mooney faces assist aspects of perceptual research [55,56]. High-contrast photographic techniques have been used to produce some of the hidden images in this article, but the colours have been introduced using computer graphics. Band pass filtering has been skilfully employed by Oliva and Schyns [57] to present different faces in the same picture. Unlike the concealed detectability of faces, the hybrid images, as they are called, are readily seen as faces, but their identities are defined by the high and low spatial frequencies they contain. The initially unseen hybrid face becomes visible when the sharply defined elements in the image are defocused or vice versa.

Despite the fact that one of the earliest random-dot-like stereograms [29] was made for the purposes of concealment, this aspect was not actively pursued until Julesz [31,32] enlisted a computer to make random-dot stereograms. Prior to that, binocular cameras produced the majority of stereoscopic images. Tyler and Clarke [28] performed a similar service for autostereograms. Binocular cooperation (stereoscopic depth) has proved more popular as a research enterprise than binocular competition (rivalry), despite the visual intrigue offered by the latter [34,58]. Anaglyphs have not been popular with scientists because the separation of the half-images to the eyes is not as precise as with optical stereoscopes. However, anaglyphs have certain advantages. The two half-images are superimposed in different colours and as such can have an allure of their own [26]. Moreover, anaglyphs produced independently can be combined in ways for which there are no equivalents for optical stereoscopes. Such combinations can be applied to both enclosed stereoscopic surfaces as well as extended ones.

Many of the illustrations in the article involve colour and will be viewed on a computer screen. The colours observed might differ from those in the original figures and might not correspond to the descriptions provided in the text. There are several reasons for this. Individual differences in colour vision have long been documented and investigated [59,60]. These differences should not influence the stereoscopic effects seen in anaglyphs because the colour filters act to block light of certain wavelengths and pass others. There is another aspect colour reproduction that could influence the colour seen—the characteristics of the computer monitor on which they are displayed [61].

## 6. Conclusions

The essence of psychophysics is to conceal a stimulus property in terms of detecting its presence or of detecting a difference between two similar stimuli. The initial examples displayed in this article mostly manipulate contrast variations in patterns. On the one hand, high-contrast patterns can use Gestalt principles of grouping to render identity difficult to discern. On the other, the interplay between the high- and low-spatial-frequency content of patterns renders the latter initially difficult to detect. The concealed aspects of the designs can be revealed by reducing the influence of the high spatial frequencies in suppressing the visibility of the low-spatial-frequency content. This can be achieved by reducing the clarity of the high-spatial-frequency content. These include blurring the pattern by defocussing (squinting or shaking the head) or by viewing the patterns from a distance. It could be argued that the revealed image is more memorable than one that is clearly distinguishable from the outset.

There are many other methods for concealing images both in two and three dimensions. One of the driving factors in the development of random-dot stereograms was the desire to present disparities without any monocular clues regarding the shape to be seen in depth. They paved the way for the investigation of stereoscopic depth perception without object recognition. Images can also be hidden, albeit briefly and dynamically, in patterns displaying binocular rivalry: radically different patterns presented to each eye visually compete in whole or parts to yield a wide variety of percepts, ranging from the dominance of one eye/pattern to intricate mosaics made up from both.

Artists have generally approached concealment from different directions—mostly through spatial ambiguity or distorted perspectives. They have also manipulated pictorial puzzles in ingenious ways. The novel illustrations of partial concealment presented in this paper can be considered as trying to bridge the gap separating visual science from visual art.

## Data Availability

No new data were created or analyzed in this study.
